# Saudi Women’s Perception of the Effect of COVID-19 Infection and Vaccination on Menstrual Cycle Length

**DOI:** 10.1089/whr.2023.0150

**Published:** 2024-06-05

**Authors:** Wael Nazzal, Thekra N. Al-Maqati, Muneera Abdulaziz Almulhim, Eman Saleh Alsulmi, Jassas F. Alotaibi, Salma AlBahrani, Omar Alsuhaibani, Eman H. Alenezi, Sattam Albusaili, Abdulelah Alharbi, Ahmed Alqahtani, Fahad Alahmari, Abdullah Alshahrani, Dhai A. Al Otaibi, Asrar H. Alfaifi, Ohood A. Madkhali

**Affiliations:** ^1^ Department of Obstetrics and Gynecology, King Fahad Military Medical Complex, Dhahran, Saudi Arabia.; ^2^ Department of Clinical Laboratory Sciences, Prince Sultan Military College of Health Sciences, Dammam, Saudi Arabia.; ^3^ Department of Obstetrics and Gynecology, King Fahad Hospital University, Imam Abdulrahman Bin Faisal University, Dammam, Saudi Arabia.; ^4^ Department of Physical Therapy, King Fahad Military Medical Complex, Dhahran, Saudi Arabia.; ^5^ Internal Medicine Department, King Fahad Military Medical Complex and Department of Internal Medicine, College of Medicine, Imam Abdulrahaman Bin Faisal University, Dammam, Saudi Arabia.; ^6^ Prince Sultan Military Medical City, Riyadh, Saudi Arabia.; ^7^ Preventive Medicine, King Fahd Military Medical Complex, Dhahran, Saudi Arabia.; ^8^ Academic Affairs, Prince Sultan Military College of Health Sciences, Dhahran, Saudi Arabia.; ^9^ Department of Pathology and Laboratory Medicine, Armed Forces Hospital, Jubail, Saudi Arabia.

**Keywords:** COVID-19, COVID-19 infection, menstrual cycle, menstrual period, vaccine

## Abstract

**Background and Aim:**

This study was conducted in the Kingdom of Saudi Arabia to investigate the effects of the COVID-19 virus and the vaccine on menstrual periods. The data from this study would increase people’s awareness of the impacts of the virus and its vaccines on menstrual periods and serve as a reference for further studies.

**Materials and Methods:**

The data was collected through a web link where standardized close-ended questionnaires were distributed *via* several social media platforms in Saudi Arabia.

**Results:**

The study included 691 respondents, with 411 women meeting the inclusion criteria. The majority of participants fell within the age range of 35–45, and 64% held at least a bachelor’s degree. The Eastern region of Saudi Arabia had the highest percentage of participants, while the Northern region had the lowest. More than half of the participants were married, and 57% reported having been infected with COVID-19. The vast majority (99%) had received the COVID-19 vaccine, primarily the Pfizer/BioNTech vaccine. The study assessed the association between menstruation experience and symptoms in three situations: before infection or vaccination, after COVID-19 infection, and after vaccination. Differences were observed in the length of the menstrual cycle and flow, but no statistically significant differences were found for pelvic and back pain.

**Conclusions:**

The result of this current study suggests that COVID-19 infection and/or vaccination has several effects on the menstrual cycle which changes in menses are minimal and transient.

## Introduction

The World Health Organization (WHO) proclaimed COVID-19 a global pandemic in March 2019. According to Chu et al., COVID-19, or coronavirus is a multisystemic infection characterized by severe respiratory distress syndrome coronavirus-2 involvement (SARS-CoV-2). Close contact between persons promotes the spread of this virus *via* respiratory secretions and aerosol particles.^1^ As a result, public health safety measures have been created globally to avoid the disease’s rapid spread. These exceptional measures included social isolation, mask-wearing, and further restrictions. This has resulted in changes in people’s daily lives with a profound impact on the population’s psychological, physical, and social well-being.^2^ To eradicate COVID-19, the WHO suggested the use of vaccines that are both safe and effective, wearing of masks, hand washing, and good ventilation indoors. The illness causes acute respiratory distress syndrome, pneumonia, renal impairment, cardiac failure, and gastrointestinal disorders.^3–6^ It affects the body’s immune system and induces a rise in interleukin (IL)−6, IL-8, tumor necrosis factor-(TNF-), as well as other cytokines.^7^

While it is reasonable to expect that there are hazards associated with any vaccine, the Centers for Disease Control and Prevention reports that serious adverse effects are extremely uncommon and that a majority of the minor side effects experienced subside after a few days. Exhaustion, general soreness, headaches, chills, and fever are among the symptoms that could occur on a systemic level. However, common adverse reactions may include swelling at the injection site, redness, and pain. The COVID-19 vaccine reduces the severity of COVID-19 by about 50%, and this has been confirmed by the Food and Drug Administration (FDA). Furthermore, the connection between COVID-19 and its vaccine with menstruation problems may cause vaccine hesitancy. On social media, many women stated that they reported experiencing premenstrual or menstrual alterations a few months following the infection or vaccination, which Alvergne et al. relate to this scenario.^8^

The official channels for reporting menstrual-related adverse events remain anecdotal. Yet, the effects of COVID-19 and its vaccine on female reproductive health, particularly the menstrual cycle, have not been collected during clinical trials. This implies that there is a need for more research to understand better the physiological processes underlying these frequently mentioned changes. Most studies focusing on the effect of COVID-19 on the menstrual cycle are literature reviews drawing from earlier work or freely accessible sources regarding the modification of ovarian reserve and sex-related hormones in COVID-19 patients.^7,9^ Some survey-based cross-sectional research have examined the effects of COVID-19 infection on the menstrual cycles and assessed how women felt about this issue. They found that blood loss and the number of days between periods fluctuated. It was also reported that the amount of blood and the time frame between periods. Therefore, these studies show that COVID-19 affects mensturation.^10,11^

Furthermore, there were limitations among studies in Saudi Arabia and the Middle East in assessing the effect of COVID-19 and its vaccine on the menstrual cycle. In the United Kingdom, the Medicines and Healthcare Products Regulatory Agency has recorded at least 36,000 cases of menstruation changes or irregularities such as unexpected vaginal bleeding after receiving COVID-19 immunization.^12^ However, the studies regarding the effect of the vaccine on menstruation found no significant differences in menstrual cycle parameters, such as cycle length and menstrual flow, after COVID-19 vaccination. The studies suggest that COVID-19 vaccination does not have a major impact on menstrual cycle parameters.^13–18^ The study observed an increased occurrence of menstrual disturbances, such as delayed menstruation, prolonged menstrual duration, heavy bleeding, and early bleeding, in women aged 18–30 years after receiving the COVID-19 vaccine. The findings indicate that there might be a greater likelihood of menstrual disturbances in this age group following vaccination.^19^ Conducted in Karachi, Pakistan, this study discovered that more than half of the women experienced changes in their menstrual cycles after getting vaccinated against COVID-19. Women reported higher rates of menstruation delay, prolonged menstrual duration, heavy bleeding, and early bleeding compared to their menstrual cycles before vaccination. The study emphasizes the impact of COVID-19 vaccination on menstrual cycle disturbances.^20^ As a result of the knowledge gap, this study in Saudi Arabia investigated the effect of the COVID-19 virus and vaccines on menstrual periods. The data from this study would increase people’s awareness of how the COVID-19 virus and its vaccines affect menstruation and serve as a reference for further studies.

## Materials and Methods

### Study design

In this study, an online survey in which standardized close-ended questionnaires were distributed to participants *via* a web link *via* various social media platforms. This study was approved by the Institutional Review Board of Prince Sultan Military College of Health Sciences (IRB-2022-CLS-046).

### Study sample (participants)

This study used a nonprobability sampling method. In this case, the target population was females from 18 to 45 years of age living in five regions in Saudi Arabia. Several biological and legal issues have contributed to the selection of this age category for women. In terms of biological factors, hormone control usually fails in women before the age of 18 and beyond the age of 45. International custom defines the age of majority as 18 for legal reasons. The exclusion criteria in the study aimed to ensure that the sample population consisted of women without certain medical conditions or factors that could potentially influence the outcomes of the study. The women were asked specific questions related to visiting the hospital because of change(s) in their menstrual cycle before COVID-19 and vaccinations or having irregular menstruation refers to a menstrual pattern that deviates significantly from the average 28-day cycle, including variations in cycle length, heavy or prolonged bleeding, or skipped periods. Also, they were asked if they were diagnosed with one of these medical conditions: Polycystic ovaries, cancer, disturbance of any gland, uterine fibroid, adenomyosis, endomteriors, and polyps and used any hormonal contraception.

### Procedures

The data used was collected through an online survey in which standardized close-ended questionnaires were distributed to participants *via* a web link *via* various social media platforms from December 2022 until the end of February 2023. The researchers, based on the Women’s Health Symptom Survey, developed the survey.^21^ The researcher also designed a structured online questionnaire based on the study’s primary objective. This online survey allowed the researcher to increase the response rate, such as emails and social media. The online surveys included an explanation of the research’s objective and significance and confirmation of the informed consent. A panel of experts validated the questions and then tested with 60 participants whose data was excluded from the final data. The test data was used to verify the questionnaires’ internal consistency using SPSS with a reliability coefficient (Cronbach’s alpha). Cronbach’s alpha for the reliability of the questionnaire was 0.7. The questionnaires were standardized to examine critical issues concerning the research question.

### Data analysis

Data collected was analyzed using the SPSS1 software version 26, with descriptive statistics being calculated as frequency and percentage. The chi-square test was used to compare the distribution of responses to various questions about women’s menstruation experience and symptoms according to the Women’s Health and Symptom Survey (WHSS) across different categories. These categories include: Before COVID-19 and vaccination, after vaccination, and after infection with COVID-19. Chi-square test was used to determine if there are significant differences in the distribution of responses among these categories where *p*-value was statistically significant when *p* < 0.05.

## Results

### Demographic characteristics

We received 691 questionnaire respondents, and five did not participate in the study. Of those who participated, 411 women were included in the study while 280 were excluded because of exclusion criteria such as a history of irregular menstruation, use of hormonal contraception, and diagnosis with polycystic ovaries. The findings in Table 1 of the study indicate that approximately half of the participants (47%) fall within the age range of 35–45. However, the lowest percentage was recorded for the 18–24 age group, which accounted for only 23% of the participants. In terms of educational status, 64% of the participants held at least a bachelor’s degree, while the lowest percentage (3%) was observed among those with a PhD. For nationality, the majority of the participants (97%) were Saudis, whereas only 3% were non-Saudi.

**Table 1.** Demographical Characteristic (*n* = 411)

Basic characteristic	Frequency (percentage)
Age group:	
18–24	93 (23%)
25–34	125 (30%)
35–45	193 (47%)
Education level:	
Under bachelor	89 (22%)
Bachelor	266 (64%)
Master	45 (11%)
PhD	11 (3%)
Nationality:	
Saudi	400 (97%)
Non-Saudi	11 (3%)
Region:	
Central region	89 (22%)
Western region	93 (23%)
Eastern region	166 (40%)
Southern region	58 (14%)
Northern region	5 (1%)
Employment status:	
Student	75 (18%)
Employed	187 (46%)
Nonemployed	149 (36%)
Marital status:	
Single	145 (35%)
Married	244 (59%)
Divorced	19 (7%)
Widowed	4 (1%)
Were you ever infected with COVID-19?	
Yes	235 (57%)
No	176 (43%)
How many times were you infected with COVID-19?	
None	176 (43%)
One time	183 (45%)
Two times	43 (10%)
More than two times	9 (2%)
Did you get the COVID-19 vaccine?	
Yes	406 (99%)
No	5 (1%)
How many doses did you get?	
None	5 (1%)
One dose	9 (2%)
Two doses	75 (18%)
Three doses	312 (76%)
Four doses	10 (3%)
Which type of vaccinations did you receive?	
Pfizer/BioNTech	273 (67%)
Modern/Spikevax	6 (2%)
Oxford/AstraZeneca	25 (6%)
Mixed	102 (25%)

When considering the geographical distribution of the survey respondents across five regions, 40% of them resided in the Eastern region of Saudi Arabia, making it the most populated region. Conversely, the Northern region had the lowest percentage of participants, with only 1% residing there. Approximately half of the participants (46%) were employed, whereas the lowest percentage (18%) consisted of students.

In terms of marital status, more than half of the participants (59%) were married, while the lowest percentage (1%) comprised widowed individuals. A majority of the participants (57%) reported having been infected with COVID-19, while 43% had not contracted the virus. Among those who were infected, approximately half (45%) experienced the infection only once, whereas the lowest percentage (2%) had been infected multiple times. The vast majority of participants (99%) had received the COVID-19 vaccine, whereas 1% had not. Among those vaccinated, the majority (76%) had received three doses, while the lowest percentage (1%) had not received any doses. In terms of vaccine type, more than half of the participants (67%) had received the Pfizer/BioNTech vaccine, whereas the lowest percentage (2%) had received the Modern/Spikevax vaccine.

### Assessment of the association of menstruation experience and symptoms among women within three periods

We compared women’s menstruation experience and symptoms in Table 2 by using the WHSS survey, which consists of four questions, among three situations: (the normal condition) before being infected or vaccinated, after COVID-19 infection, and after getting the vaccination. In the normal condition category, all 411 participants responded to the survey questions, but 235 responded to the questions related to the condition or situation of “after being infected.” Similarly, 406 participants responded to the questions related to the situation of “after being vaccinated.” When it comes to the length of the period cycle, we compared the three groups, Table 2 shows that 29% of the participants said that their periods were lasting less than 21 days before the infection of COVID-19. Another 34% of the participants said that their periods lasted less than 21 days after the infection of COVID-19. In addition, about 33% of the participants, compared with the normal condition, said that their periods lasted less than 21 days after the vaccination. When we compared the length of period time between the three groups, we found an increase in the number of days (more than to say and less than to say), so since the *p*-value < 0.005, there are statistically significant differences. Regarding the menstrual flow, most participants (82%) said that their period’s flow was moderate before the infection of COVID-19. More than half of the participants (53%) said that their period flow was moderate after the infection of COVID-19. A further 55% of the study participants said their period’s flow was moderate after the vaccination. Since the *p*-value is < 0.005, it points to the existence of statistically significant differences.

**Table 2.** Comparing Menstruation Experience and Symptoms of Women *via* Chi-Square

WHSS	Normal condition before infection and vaccination (*n* = 411)	After Covid infection (*n* = 235)	After vaccination (*n* = 406)	*p*-value
How long is your period cycle?				
Less than 21 days	119 (29%)	79 (34%)	134 (33%)	<0.0001^a^
22–24 days	98 (24%)	53 (23%)	77 (19%)	
25–28 days	107 (26%)	64 (27%)	78 (19%)	
29–32 days	44 (10%)	0	37 (9%)	
33–35 days	15 (4%)	0	21 (5%)	
More than 36 days	2 (1%)	7 (3%)	5 (1.2%)	
Too irregular to say	26 (6%)	32 (13%)	54 (13%)	
How is your menstrual flow?				<0.0001^a^
Light	46 (11%)	61 (26%)	93 (23%)	
Moderate	337 (82%)	125 (53%)	224 (55%)	
Heavy (clots/flooding)	28 (7%)	49 (21%)	89 (22%)	
How often do you have pelvic pain?				0.837
Never	97 (24%)	57 (24%)	92 (23%)	
Occasionally (less than a quarter of the time)	173 (42%)	77 (33%)	151 (37%)	
Often (a quarter to half of the time)	76 (19%)	45 (19%)	81 (20%)	
Usually (more than half of the time)	34 (8%)	38 (16%)	52 (13%)	
Always (every time)	31 (8%)	18 (8%)	30 (27%)	
How often have you had back pain?				0.436
Never	50 (12%)	36 (15%)	56 (14%)	
Occasionally (less than a quarter of the time)	171 (42%)	79 (34%)	143 (35%)	
Often (a quarter to half of the time)	88 (21%)	46 (20%)	93 (23%)	
Usually (more than half of the time)	61 (15%)	43 (18%)	65 (16%)	
Always (every time)	41 (10%)	31 (13%)	49 (12%)	

^a^ Statistically significant.

For pelvic pain, about half of the participants (53%) said that they always had pelvic pain before the infection of COVID-19, while 8% said that they always had pelvic pain after the infection of COVID-19. Another 27% of the participants pointed out that they always had pelvic pain after being vaccinated. Since the *p*-value is > 0.005, no statistically significant differences were found between the responses. For the back pain, almost half of the participants (42%) said that they occasionally had pelvic pain before the infection of COVID-19 while 34% reported that they occasionally had pelvic pain after the infection of COVID-19. Lastly, 35% of the participants said that they occasionally had pelvic pain after being vaccinated. Since the *p*-value is > 0.005, it implies that no statistically significant differences exist between the responses.

## Discussion

When it comes to women’s health, the menstrual cycle ranks high on the list of essential physiological functions. It is a complex hormonal process that can be influenced by things like diet, sleep patterns, stress levels, and even drugs. For most females, it is a natural process occurring in the reproductive years, *i.e.*, the period between puberty and menopause. Studies have shown that a normal cycle lasts between 22 and 35 days (about 1 month 4 and a half days).^8^ It is considered an indicator of women’s quality of life and reproductive health. The menstrual cycles include changes in menstrual volume, rhythm, frequency, and duration, as well as changes in premenstrual syndrome and increased complaints of dysmenorrhea.^9^ Women who experience menstrual issues may have to seek medical attention more frequently and this may reduce their quality of life.^15,18,19^ Still, vaccine trials have not included questions and concerns about post-vaccination menstruation effects. As a result, the effect of immunization on women is still unclear. Different aspects, for instance, the prevalence of menstrual abnormalities, the duration of these changes, and the predicted oscillations in menstrual cycles are currently under-researched.^20^ According to existing research, some of the most cited adverse side effects of the COVID-19 vaccine include myalgia, fatigue, fever, and pain.^22^ However, it is worth noting that the rates and intensity of these effects differ from person to person. Scholars^23–25^ have argued that they may last for a few days in some individuals but longer for others. Similarly, some COVID-19 vaccines cause more side effects following the initial dose, whereas others do so after successive doses.

Consequently, the results of this study have revealed several things concerning the relationship between COVID-19 infection and its vaccines and the menstrual cycle. First and foremost, it has shown significant variations in the period cycle length. Second, there are significant differences between the responses in the flow of the period cycle. However, it failed to find significant differences between pelvic and back pain.^25^ Third, the result showed changes in the menstrual cycles both during and after COVID-19 infection on various aspects, including the quantity of blood, the length of menses, as well as the duration of time between successive cycles. This can be caused by psychological disorders, such as sadness and severe stress resulting from the COVID-19 infection.^26^

When it comes to the length of menses, this research has shown that the length of the menstrual cycle increased during infection compared to before the infection. These findings are similar to the study that reported that they had light menstrual flow increased two times, the premenopausal women who had moderate menstrual flow decreased by 29% after infection and 27% after vaccination and those who had heavy menstrual flow increased by near to threefold.^25^ These results indicate that the menstrual flow can become more abnormal and irregular, either light or heavy, due to COVID-19 infection and/or vaccination.^24^ These findings corroborate Trogstad who found that vaccination causes a change in the flow of menstruation, as he found that the menstruation was heavier-than-usual bleeding.^19^ This result coincides with what was stated in Merchant, where post-vaccination anomalies in women’s menstrual cycles, including heavy menstrual flow and frequent menstrual flow, have been reported, as have postmenopausal flow and heavy periods (metrorrhagia/polymenorrhea).^23^ Merchant concluded that vaccine-induced thrombocytopenia is an underlying cause. However, stress, weight gain, hormones, and other factors can affect the menstrual cycle.

This was also confirmed by the Medicines and Healthcare Products Regulatory Agency following many reports of irregularities in the menstrual cycle following vaccination against the coronavirus.^12^ The findings of the current study also corroborate that of Cohort who found that COVID-19 and vaccination caused changes and irregularities in the length of the menstrual cycle.^18^ They also agree with male study which reported that the number of people reporting menstrual cycle changes after COVID-19 immunization is growing.^14^ The results agree with Sualeh and her colleagues who found menstruation abnormalities after receiving a vaccination, and Muhaidat and others who stated that many women around the world have reported experiencing irregular menstrual cycles following the COVID-19 immunization since its introduction.^20,23,24^Al-Najjar and others found that COVID-19 infection can affect the menstrual cycle.^10^ However, the current study’s results differ from those found by Baena-García and her colleagues that none of the SARS-CoV-2 vaccination clinical trials indicated menstruation problems as a potential side effect.^15^

For the Saudi studies, the current aligns with the findings of Qashqari and her colleagues who identified a potential relationship between COVID-19 vaccines and irregularities of the menstrual cycle, with negative effects on the quality of life for women.^25^ This is corroborated by Alahmadi and others whose study showed that COVID-19 immunization caused a brief disruption in the menstrual cycle, manifested primarily by increased menstrual discomfort and bleeding.^27^

This current study investigated the changes in pelvic pain due to COVID-19 infection and/or vaccination. The frequency of premenopausal women who reported that they usually suffer from pelvic pain increased after COVID-19 vaccination with 27%. An earlier study was conducted at the University of Khartoum on the same issue and found a significant link between COVID-19 vaccination and increased back pain during the period.^28^ From the study’s findings, a direct comparison between COVID-19 vaccinated and nonvaccinated participants revealed that vaccinated females had a higher frequency of back pain than their unvaccinated counterparts.^29^ However, the current study does not match this study, and we noticed that the back pain did not significantly increase or be related to COVID-19 infection and/or vaccination.

In this study among our sample of Saudi women asked to recall menstrual cycle characteristics, their perception was that cycles were more irregular after COVID-19 infection and vaccination. These findings highlight the significance of ensuring a person’s physical and emotional well-being during an outbreak to safeguard their reproductive health whether in the case of COVID-19 or other coming pandemics.

However, the current study has several imitations. First, a limitation arises from the fact that it employed a cross-sectional study design which prevented any conclusions concerning the effects of COVID-19 infection and/or vaccination on the female reproductive system, particularly the menstrual cycle. Second, because the study adopted online data collection and convenience sampling, this limits the generalizability of the results online only, and not all women have online access. The population of some regions is more than twice the population of some other regions and the percentage of the employment status of Saudi women is not representative of what has been reported in Saudi Women Report 2022 as the unemployment of Saudi women was 15.4%. in addition, the age group depends on the Saudi Women Report 2022 reported that the highest age group from 18 to 24 and the lowest number from 35 to 45 which implies that the sample was not fully representative of the entire Kingdom. Also, our study is based on recall where data, in which the data is not current may result in bias due to imperfect recall. Note that the 29% rate of having a period duration of less than 21 days in the “normal condition before infection and vaccination” is much higher than expected and unlikely to be reliable.

## Conclusions

The COVID-19 pandemic is one of the most serious health crises and has a significant impact on women’s health. Among the adverse side effects reported by individuals include sore arms, headaches, and fevers. According to the studies highlighted in this article, these are signs that the body’s mounting an immune response and learning how to fight the coronavirus. Nevertheless, the growing interest and concern among women of reproductive age have pointed to a gap in how clinical research on the COVID-19 vaccines has been conducted. Like other vaccine trials, very few previous studies on the COVID-19 pandemic have given much consideration to reproductive health aside, particularly how COVID-19 and the developed vaccines affect the menstrual cycle. The current study has shown that COVID-19 infection leads to many changes in women of reproductive age. Such changes can adversely impact the menstrual cycle, including the quantity of blood, length of menses, and its irregularity. It has corroborated earlier studies that have found that following infection and vaccination, many women experienced heavy flows or bleeding at an unexpected time in the menstrual cycle, also referred to as menstrual spotting or breakthrough bleeding. Consequently, the adverse changes may severely affect a woman’s reproductive health.

## Ethic Committee

This study’s approval was obtained from the Institutional Review Board of Prince Sultan Military College of Health Sciences (IRB-2022-CLS-046).

## Notes

### Abbreviation Used

ARDS: acute respiratory distress syndrome
CDC: the Centers for Disease Control and Prevention
COVID-19: Coronavirus disease 2019
IL-6: interleukin 6
IL-8: interleukin 8
SARS-CoV-2: severe-acute-respiratory-syndrome-related coronavirus-2
WHO: World Health Organization
TNF: tumor necrosis factor
FDA: the Food and Drug Administration
WHSS: Women's Health and Symptom Survey
